# Effect of Family Caregiver Need for Training on Medicare Home Health Care

**DOI:** 10.1093/geroni/igaa057.488

**Published:** 2020-12-16

**Authors:** Julia Burgdorf, Elizabeth Stuart, Jennifer Wolff

**Affiliations:** Johns Hopkins Bloomberg School of Public Health, Baltimore, Maryland, United States

## Abstract

Medicare home health providers are required to offer family caregiver training; however, there is little information regarding the impact of family caregiver training on home health care intensity. A better understanding of this relationship is necessary to inform development and prioritization of caregiver training interventions in this setting. This research assesses whether and how family caregiver need for training affects care intensity during Medicare home health. We examine 1,217 (weighted n=5,870,905) fee-for-service Medicare beneficiaries who participated in the National Health and Aging Trends Study (NHATS) between 2011-2015 and received Medicare-funded home health care within one year of survey. Using propensity score adjusted, multivariable logistic and negative binomial regression, we model the relationship between family caregiver need for activity-specific training and the number/type of visits received during Medicare home health. We found that older adults whose family caregiver required training on self-care tasks had greater odds of receiving any therapy visits (aOR: 1.70; 95% CI: 1.01, 2.86), aide visits (aOR: 2.12; 95% CI: 1.11, 4.05), or training visits (aOR: 1.49; 95% CI:1.01, 2.21). Older adults whose family caregiver required training on medication management had greater odds of receiving any nursing visits (aOR: 3.03; 95% CI: 1.06, 8.68) and incurred 1.06 (95% CI: 0.11, 2.01) additional nursing visits. Findings support the importance of connecting family caregivers to training resources. Additionally, findings suggest that home health providers should consider prioritizing training interventions which focus on caregiving activities most closely tied to resource utilization: self-care and medication management.

